# Investigation of RNA Viruses in *Culicoides* Latreille, 1809 (Diptera: Ceratopogonidae) in a Mining Complex in the Southeastern Region of the Brazilian Amazon

**DOI:** 10.3390/v16121862

**Published:** 2024-11-29

**Authors:** Sâmia Luzia Sena da Silva, Sandro Patroca da Silva, Carine Fortes Aragão, Inocêncio de Sousa Gorayeb, Ana Cecília Ribeiro Cruz, Daniel Damous Dias, Bruna Laís Sena do Nascimento, Jannifer Oliveira Chiang, Lívia Medeiros Neves Casseb, Joaquim Pinto Nunes Neto, Lívia Carício Martins, Pedro Fernando da Costa Vasconcelos

**Affiliations:** 1 Department of Arbovirology and Hemorrhagic Fevers, Evandro Chagas Institute, Secretariat of Health and Environment Surveillance, Ministry of Health, Ananindeua 67030-000, PA, Brazil; samialuzia23@gmail.com (S.L.S.d.S.); sandrosilva@iec.gov.br (S.P.d.S.); carinefaragao@gmail.com (C.F.A.); anacecilia@iec.gov.br (A.C.R.C.); danieldias@iec.gov.br (D.D.D.); brunanascimento@iec.gov.br (B.L.S.d.N.); janniferchian@iec.gov.br (J.O.C.); liviacasseb@iec.gov.br (L.M.N.C.); joaquimneto@iec.gov.br (J.P.N.N.); liviamartins@iec.gov.br (L.C.M.); 2 Coordination of Zoology, Entomology, Museu Paraense Emílio Goeldi, Belém 66077-830, PA, Brazil; gorayeb@museu-goeldi.br; 3 Centre for Biological and Health Sciences, Pará State University, Belém 66087-662, PA, Brazil

**Keywords:** *Culicoides*, biting midges, metagenomic, virome, viruses, insect specific-viruses

## Abstract

The biting midges *Culicoides* Latreille, 1809 (Diptera: Ceratopogonidae) is highly relevant to epidemiology and public health, as it includes species that are potential vectors of human and animal arboviruses. The aim of this study was to investigate the presence of RNA viruses in species of the genus *Culicoides* collected in the Carajás mining complex in the state of Pará. The biting midges were collected in the municipalities of Canaã dos Carajás, Curionópolis and Marabá and morphologically identified. A total of 1139 specimens of seven *Culicoides* species were grouped into eight pools and subjected to metagenomic analysis. Eight new insect-specific viruses (ISVs) were characterized and assigned to the order *Tolivirales*, the families *Chuviridae*, *Nodaviridae*, *Iflaviridae*, *Mesoniviridae*, and *Flaviviridae,* and the taxon *Negevirus*. All viruses identified were assigned to clades, families and taxa never reported in *Culicoides* in Brazil. This study demonstrated that biting midges harbor a significant diversity of RNA viruses, many of which are still unknown, highlighting the importance of studies aiming at virome of these insects.

## 1. Introduction

Viruses can infect practically all forms of life and are only maintained in nature if they can be transmitted from one host to another, whether of the same species or not [1]. Even with the recent re-emergence of several arboviruses, it is estimated that less than 1% of all viruses have been discovered [2].

For a long time, the strategies for discovering new viruses were based on classical technologies such as electron microscopy, cell culture, and serological methods. Although these approaches have led to the discovery of various agents, they present methodological limitations [3].

The emergence of molecular methods has brought substantial changes to virological research since the genetic material of a virus became accessible and identifiable [4]. New sequencing strategies, such as “viral metagenomics”, consist of studying the nucleic acid sequences of non-cultivable viruses from different biomes, which has enabled the knowledge of an extraordinary and unimaginable viral diversity, with hundreds of new viruses described annually [5,6,7].

Metagenomic studies often focus on arthropod species of economic and medical importance, such as bees and vector mosquitoes [8,9,10]. Among vector arthropods, which primarily include mosquitoes (Culicidae), ticks (Ixodidae), sandflies (Psychodidae, Phlebotominae), black flies (Simuliidae), and biting midges (Ceratopogonidae), biting midges have received less attention regarding their ability to harbor and transmit viruses, despite being the most abundant blood-feeding insects worldwide [11].

Within the family Ceratopogonidae, the genus *Culicoides* Latreille, 1809 includes species that have medical and veterinary importance, with a diversity of viruses already isolated in various species. Most of the viruses isolated from *Culicoides* species belong to the families *Peribunyaviridae*, *Sedoreoviridae*, and *Rhabdoviridae* [11,12,13,14,15,16].

Modha et al. [17] noted the absence of metagenomic studies on *Culicoides* species and considered the description of the virome of these arthropods relevant for assessing future risks associated with them. In their study, these authors detected a series of nearly complete genomes, as well as partial genomes of new viruses in *Culicoides impunctatus* Goetghebuer, 1920, a species that is a source of nuisance bites to humans and a vector of avian malaria in Scotland [17]. Another similar study compared the viromes of *Culicoides* and mosquitoes in Yunnan Province, China, and revealed that, in the same locations, these insects generally share a similar viral diversity. The findings of this study also suggested that *Culicoides* might play a more significant role than expected in viral ecology and disease transmission and should be considered important targets for the monitoring, prevention, and control of infectious diseases [18].

Although *Culicoides* is known to include potential vectors of infectious agents, studies investigating viruses in species of this genus are rare in Brazil. In the Amazon region, the epidemiological studies on the *Orthobunyavirus oropoucheense* stand out, which only record the species *Culicoides paraensis* Goeldi, 1905 as the primary vector of this virus [19,20]. The re-emergence and geographic expansion of *Orthobunyavirus oropoucheense* into previously non-endemic areas between 2023 and 2024, with an increase in cases of Oropouche Fever, as well as the possibility of vertical transmission and the report of two deaths caused by the disease, something never documented in the global scientific literature [21,22,23], highlight the need to develop studies aimed at understanding the virome of *Culicoides* species.

To date, no published studies have been found using metagenomics to investigate the virome of ceratopogonids identified at the species level. Therefore, the objective of this study was to investigate the presence of RNA viruses in species of the *Culicoides* genus collected in the Carajás mining complex area in the southeastern region of the state of Pará, in the Brazilian Amazonia.

## 2. Materials and Methods

### 2.1. Study Area

**Figure 1:**
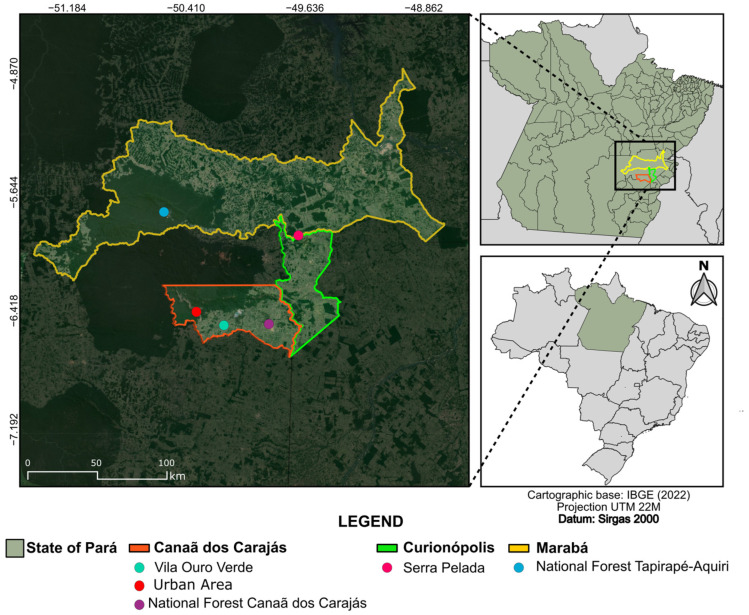
Map showing the collection points of ceratopogonids in the municipalities of Canaã dos Carajás, Curionópolis, and Marabá, located in the state of Pará, Brazilian Amazonia.

This study was conducted in the localities of Vila Ouro Verde (S 06°31′38.2″; W 50°08″52.5″), an urban area (S 06°31′08.1″; W 49°51′18.8″), and Carajás National Forest (S 06°26′24.8″; W 50°19′37.2″), all located in the municipality of Canaã dos Carajás; Serra Pelada (S 05°56′35.3″; W 049°40′38.7″) in the municipality of Curionópolis; and Tapirapé-Aquiri National Forest (S 05°46′16.4″; W 50°33′15.8″) in the municipality of Marabá, in the state of Pará, in the Amazonia region, Brazil (Figure 1). These municipalities are within the area of influence of the Carajás mining complex. The activities were authorized by the Chico Mendes Institute for Biodiversity Conservation (ICMBIO), under authorization numbers 021-2018 and 57-2019.

### 2.2. Collection and Identification of Biting Midge Samples

Biting midge collections were carried out in February, March, and April 2019, over six consecutive days. The midges were captured using CDC (Centers for Disease Control and Prevention) light traps, set 1.5 m above ground level in the environments selected for the study. The traps remained operational from 6:00 p.m. to 6:00 a.m. the following day. The specimens were transported in liquid nitrogen to the laboratory, where the identification of morphotypes was conducted on a refrigerated table using a stereoscopic microscope. The midges were grouped based on similarities in their observed morphological characteristics. For species-level identification, some specimens were mounted on slides using the phenol-balsam method [24] with adaptations by Felippe-Bauer (FIOCRUZ-RJ). After mounting, the specimens were identified using identification keys and specialized literature. Once identified, the specimens with the same morphotype were counted and grouped into pools, considering the species, collection locality, and collection period.

### 2.3. RNA Purification

For the extraction of genetic material, each pool was macerated in 1 mL of Dulbecco’s Phosphate-Buffered Saline (DPBS) solution, supplemented with fetal bovine serum, antibiotics, and antifungal agents, using a 3 mm tungsten bead and the TissueLyser II equipment for 2 min at a frequency of 25 Hz. The material was then centrifuged at 12,000× *g* at 4 °C for 10 min, and 140 µL of the supernatant was recovered for RNA purification using the QIAamp^®^ Viral RNA Mini Kit (Qiagen, Hilden, Germany), following the manufacturer’s protocol. After purification, the RNA was quantified using the Qubit RNA HS Assay Kit on Qubit 4.0 equipment, according to the manufacturer’s recommendations.

### 2.4. Double-Stranded cDNA Synthesis

From the extracted RNA, the first and second strands of cDNA were synthesized using the SuperScript^TM^ VILO^TM^ MasterMix and NEBNext^®^ Second Strand Synthesis Module kits, respectively. After synthesis, the cDNA was purified using the PureLink^®^ PCR Purification Kit and quantified with the DNA HS Assay Kit on the Qubit 4.0 equipment.

### 2.5. Genomic Library Construction and Sequencing

The genomic library was constructed using the Nextera XT DNA kit (Illumina, San Diego, CA, USA), according to the manufacturer’s instructions, and purified using AMPure XP beads. Subsequently, the library was qualitatively assessed using the Agilent High Sensitivity DNA Kit and Bioanalyzer 2100 equipment and quantitatively assessed using the DNA HS Assay Kit on the Qubit 4.0.

The library was loaded onto the NextSeq 500 sequencer (Illumina) using the NextSeq 500/550 High Output Kit v.2.5 (300 Cycles) and was sequenced by a paired-end methodology.

### 2.6. Data Analysis and Processing

The quality of the reads generated during sequencing was initially evaluated using the fastp v.0.23.2 software, with the removal of short reads (less than 50 nt), adapter fragments, and reads with indeterminate bases (reads with more than 10% N). Next, the SortMeRNA v.2.1 [25] software was used to remove ribosomal RNA (rRNA). After processing, a comparative analysis using fastp was applied to assess the data processing efficiency. Subsequently, the DIAMOND v.2.0.15 [26] software was used for the read annotation, using the non-redundant protein database (nr) for viruses, considering an e-value of 1e-4 and amino acid identity.

The output files generated by DIAMOND were converted into m8 format files, which were visualized in Krona v.2.8 [27]. The results were tabulated and saved in a CSV file, and these tables were loaded and evaluated in the R software v.4.3.3 using the ComplexHeatmap library [28] to observe the abundance patterns of the different viruses found in each sequenced sample. The Kraken program was also used, employing the same nr database, with results visualized in Pavian.

### 2.7. De Novo Assembly

The files generated during the processing step were used for assembly by de novo methodology using the SPAdes v.3.13.1 [29] and MEGAHIT v.1.2.9 [30] programs, using k-mer values of 21, 33, 55, and 77 and 21, 31, 41, 51, 61, 71, 81, 91, and 99, respectively. The contigs generated were analyzed with the BLASTX using DIAMOND program v.2.1.9 [26], using the viral protein nr database, considering e-values of 1 × 10^4^ and amino acid identity.

The files corresponding to the contigs generated for each pool were visualized using the Megan v.6.21.1 software [31], applying an e-value filter of 1 × 10^10^. Contigs showing similarity to viral sequences of interest were analyzed using Geneious v.9.1.8 software (https://www.geneious.com/, accessed on 14 February 2024).

### 2.8. Phylogenetic Analysis

Phylogenetic inference was performed using nucleotide sequences of different virus strains available from the National Center for Biotechnology Information (NCBI) database (http://www.ncbi.nlm.nih.gov, accessed on 14 February 2024), using the coding regions of the proteins.

The dataset generated, along with the samples from the study, were subjected to Multiple Sequence Alignment (MSA) using the MAFFT v.7 [32] software, and the alignment was manually inspected for corrections when necessary, using the Geneious v.9.1.8 software (https://www.geneious.com/, accessed on 14 February 2024).

Initially, the aligned dataset was analyzed to identify the best nucleotide substitution model, followed by phylogenetic tree construction using the Maximum Likelihood (ML) methodology [33]. Both methodologies were applied using the IQ-TREE v.1.6.12 software. Additionally, a bootstrap test with 1000 replicates was used to ensure the greater reliability of the clustering values [34].

Phylogeny visualization was performed with the FigTree v.1.4.4 software (https://github.com/rambaut/figtree/releases, accessed on 20 February 2024). For the dataset used, no root sequence (root) was applied; therefore, the Midpoint Rooting method was used, a tool available in the phylogeny visualization program. After phylogeny evaluation and editing, an “.sgv” (Scalable Vector Graphics) file was generated for editing and image manipulation using Inkscape v.1.1 software (https://inkscape.org/release/inkscape-1.1/, accessed on 12 March 2024).

### 2.9. Identification of Protein Domains

After the identification of phylogenetically related viruses, the translated sequences were subjected to a search for conserved protein domains using the InterProScan tool v. 5.69-101.0 (https://www.ebi.ac.uk/interpro/search/sequence/, accessed on 21 March 2024), utilizing the UniProtKB protein database.

## 3. Results

A total of 1139 specimens from seven species of the genus *Culicoides* were grouped into eight pools for sequencing. After sequencing the samples, a total of 373,894,200 paired-end reads were obtained, which were subjected to quality control procedures, reducing the amount of data to 248,392,951 reads (Table 1). Table 2 provides information on the eight genomes recovered from the *Culicoides* samples, as well as the results of comparisons with the NCBI non-redundant (nr) database using BLASTX.

### 3.1. Virome Analyses

**Figure 2:**
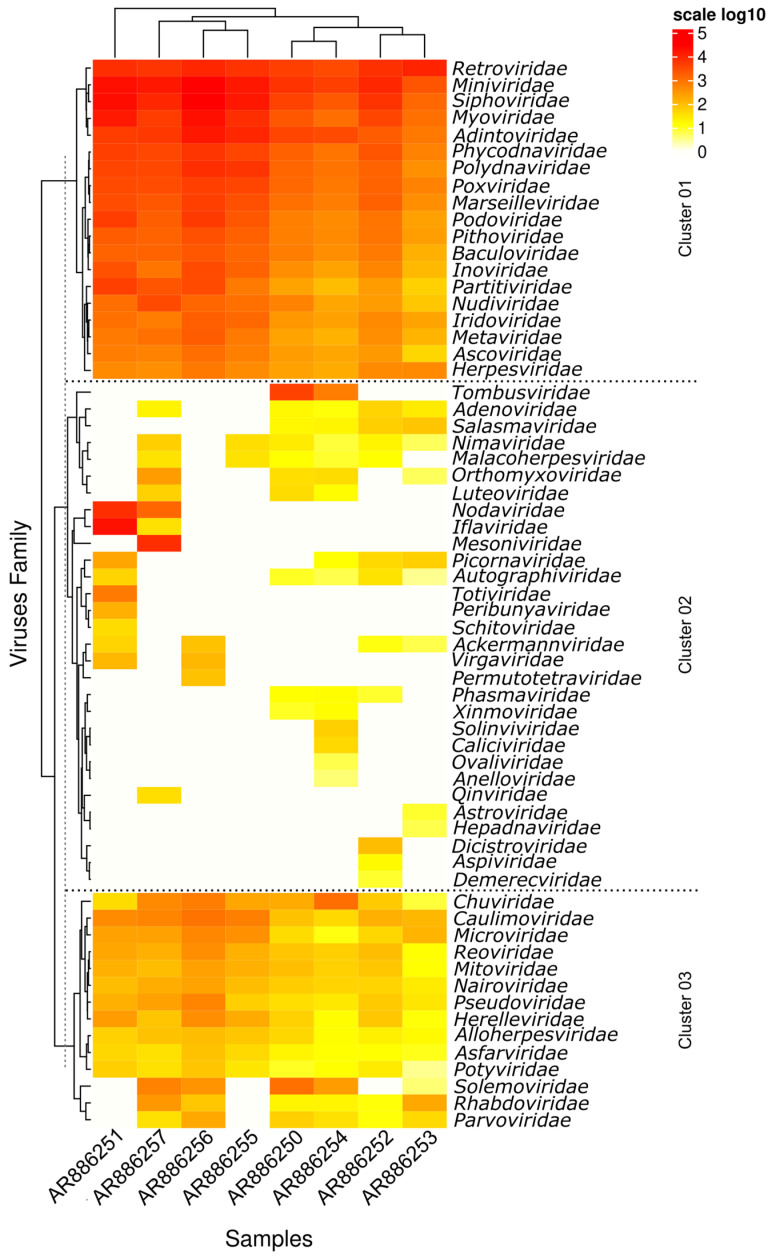
Distribution of reads of viral families found in *Culicoides* samples. The side bar corresponds to the log10 values of the total reads for each sample.

It was possible to identify different viral families that showed some level of amino acid identity with the reads obtained in this study for the various pools of *Culicoides* sequenced. However, three clusters were differentiated with varying abundances of reads (Figure 2).

In cluster 1, a higher number of reads was observed for all pools, with the families *Retroviridae*, *Mimiviridae*, *Siphoviridae*, *Myoviridae*, and *Adintoviridae* presenting the highest numbers of reads. In contrast, cluster 3 showed a lower number of reads for almost all pools, except for the families *Solemoviridae*, *Rhabdoviridae*, and *Parvoviridae*, which had no reads in pools AR886251 and AR886255, and the family *Solemoviridae*, which had no reads in pool AR886252.

In cluster 2, a lower diversity of viral identities was observed in some specific pools. Pool AR886251 presented a higher number of reads with identity to the families *Nodaviridae* and *Iflaviridae*, pool AR886257 for the family *Mesoniviridae*, and pools AR886250 and AR886254 for the family *Tombusviridae*.

Nine genomes corresponding to eight probable new viruses were recovered from four samples (AR886250, AR886251, AR886254, AR886257) of three species of *Culicoides*. These genomes are related to the order *Tolivirales* and the families *Chuviridae*, *Nodaviridae*, *Iflaviridae*, *Mesoniviridae*, and *Flaviviridae*, as well as to the taxon *Negevirus*.

#### 3.1.1. Tolivirales

Two genomes of a probable new virus associated with the order *Tolivirales* were identified from two pools of *Culicoides leopoldoi* (AR886250; AR886254) and named *Ouro verde tombus-like virus 1* (GenBank: PQ266788 and PQ266789, respectively).

The first genome had a coverage of 78×, a length of 2163 nucleotides, and two Open Reading Frames (ORFs) encoding a hypothetical protein and the non-structural protein RNA-dependent RNA Polymerase (RdRp). The second genome has a coverage of 74×, a length of 1666 nucleotides, and a single ORF encoding the RdRp protein. However, it was not possible to recover the region of the hypothetical protein in the AR886254 sample.

The BlastX analyses showed that the ORF encoding the RdRp gene of both recovered genomes presented 40.8% amino acid identity with the RdRp of *Hymenopteran tombus-related virus* (GenBank: QTJ63591.1), classified in the Gopeviruses clade (Table 2).

**Figure 3:**
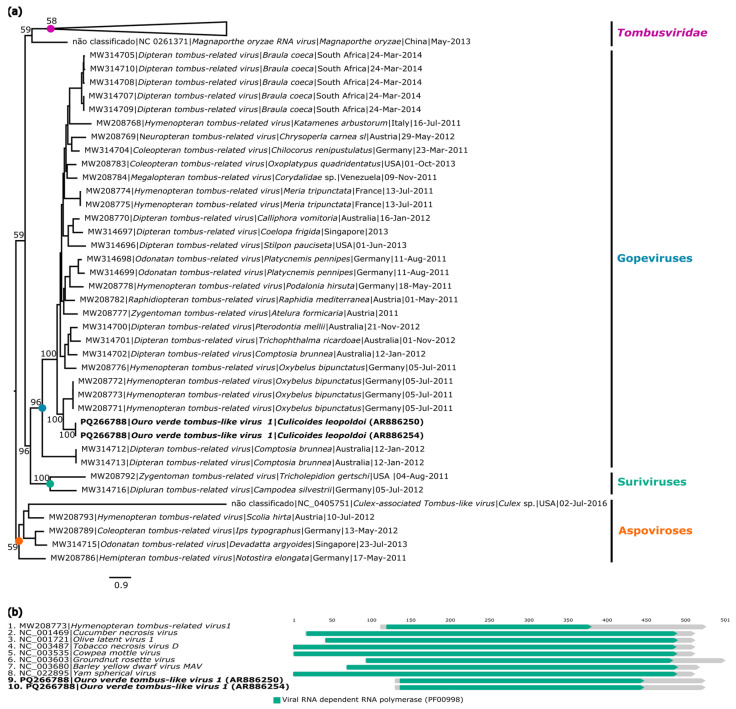
Phylogenetic relationship and genomic organization of *Ouro Verde tombus-like virus 1*. (**a**) Phylogenetic inference was performed using the Maximum Likelihood (ML) method based on the amino acid sequences of the RNA-dependent RNA Polymerase (RdRp) protein, using the LG+F+R6 matrix as the best nucleotide substitution model, and measurement of the phylogenetic signal in the dataset, showing only 33% unresolved quartets and 67% resolved quartets. Different groups are identified by different colors. The samples identified in this study are highlighted in bold. The numbers at each main node of the tree correspond to bootstrap values in percentages (1000 replicates). The scale bar corresponds to amino acid divergence per site between sequences. (**b**) Domains are displayed as colored boxes, and the sequence size is shown as the number of nucleotides.

Phylogenetic inferences were performed using the amino acid sequence of the RdRp region from the two recovered strains of *Ouro verde tombus-like virus 1*, representatives of the family *Tombusviridae* and the Gopeviruses, Suriviruses, and Aspoviruses clades. The analyses showed that the two recovered genomes are not classified in any of the genera of the family *Tombusviridae* but formed a monophyletic clade related to viruses classified in the Gopeviruses clade (Figure 3a).

The identity matrix generated from the alignment of nucleotide and amino acid sequences of the RdRp region of *Ouro verde tombus-like virus 1* (AR886250) and *Ouro verde tombus-like virus 1* (AR886254) showed that they share 98.9% nucleotide identity and 100% amino acid identity with each other, indicating that these sequences correspond to the same viral species. Moreover, both viruses showed closer proximity to the *Hymenopteran tombus-related virus* (GenBank: MW208773), with 54.8% (nt) and 49.9% (aa) identity, indicating that the two found sequences correspond to a new virus (Supplementary Table S1). Analyses obtained through the InterProScan tool identified functional protein domains common to other viruses within the Gopevirus clade (Figure 3b).

#### 3.1.2. Chuviridae

A closely related genome to the family *Chuviridae*, named *Carajas chuvirus* (GenBank: PQ226781), was identified in *C. leopoldoi* (AR886254). The recovered genome showed 53.4× coverage, is 5827 nt long, and consists of an ORF (5492 nt) located in segment L that encodes the RdRp protein.

BlastX analyses showed that the identified genome exhibited 58.3% amino acid identity with *Chuviridae* sp. (GenBank: UCR92571.1), classified in the family *Chuviridae*, referring to the region that encodes RdRp (Table 2).

**Figure 4:**
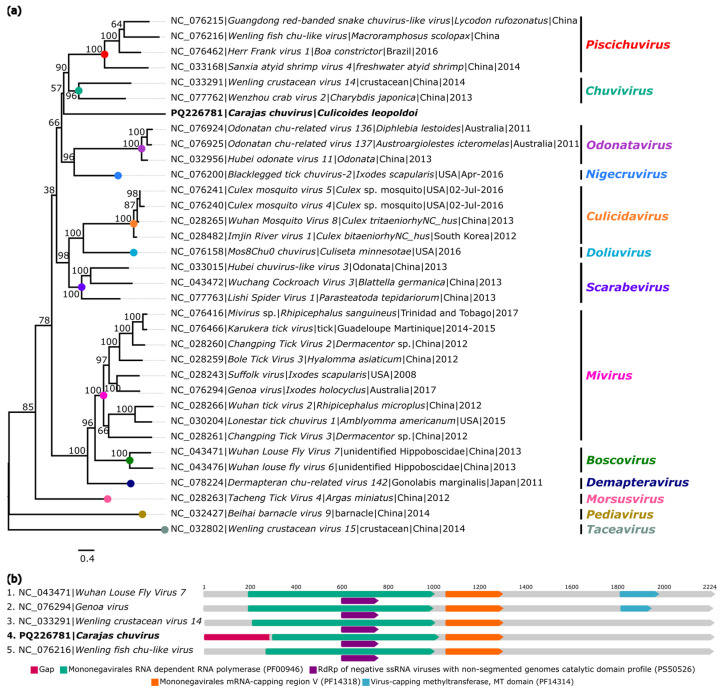
Phylogenetic relationship and genomic organization of *Carajas chuvirus*. (**a**) Phylogenetic inference was performed using the Maximum Likelihood (ML) method based on the amino acid sequences of the RNA-dependent RNA Polymerase (RdRp) protein, using the LG+F+R6 matrix as the best nucleotide substitution model, and measurement of the phylogenetic signal in the dataset, showing only 11.8% unresolved quartets and 88.2% resolved quartets. The genera are identified by different colors on the right side of the tree. The samples identified in this study are highlighted in bold. The numbers at each main node of the tree correspond to bootstrap values in percentages (1000 replicates). The scale bar corresponds to amino acid divergence per site between sequences. (**b**) Domains are displayed as colored boxes, and the sequence size is shown as the number of nucleotides.

In the phylogenetic inference, *Carajas chuvirus* did not fit within any described genus in the family *Chuviridae*. However, it formed an external branch more closely related to the genera *Piscichuvirus* and *Chuvivirus* (Figure 4a).

The low nucleotide and amino acid identity with other closely related viruses suggests that the obtained RdRp sequence corresponds to a new virus. The identity matrix (Supplementary Table S2) shows that the highest genomic correspondence was observed with *Hubei chuvirus-like virus 1* (GenBank: NC_033327), sharing 51% (nt) and 27.3% (aa). Analyses obtained using the InterProScan tool identified the same functional domains present in species of the family *Chuviridae* (Figure 4b).

#### 3.1.3. Nodaviridae

The genome of a probable new virus associated with the family *Nodaviridae* was identified from a batch of *C. insignis* (AR886251) and was named *Ouro Verde nodavirus* (GenBank: PQ226787). This genome exhibited 803× coverage, a length of 3213 nt, and an ORF designated as ORF1a that encodes the RdRp protein.

Analyses conducted with BlastX revealed that the identified genome has 51.1% amino acid identity with *Carano virus* (GenBank: BCG55383.1), a member of the family *Nodaviridae*, specifically in the region encoding RdRp (Table 2).

**Figure 5:**
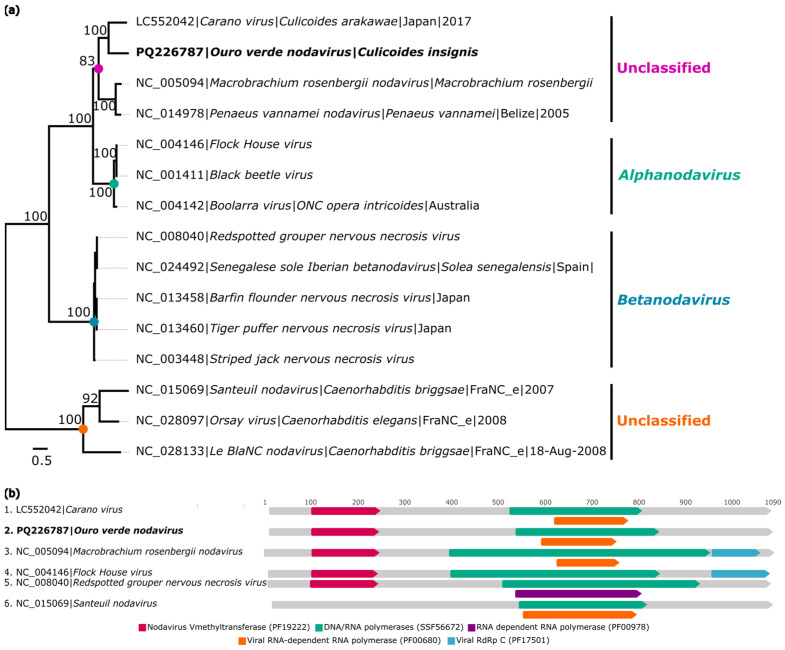
Phylogenetic relationship and genomic organization of *Ouro Verde nodavirus*. (**a**) Phylogenetic inference was performed using the Maximum Likelihood (ML) method based on the amino acid sequences of the RNA-dependent RNA Polymerase (RdRp) protein, using the LG+F+I+G4 matrix as the best nucleotide substitution model, and measurement of the phylogenetic signal in the dataset, showing only 11.4% unresolved quartets and 88.6% resolved quartets. The genera are identified by different colors on the right side of the tree. The samples identified in this study are highlighted in bold. The numbers at each main node of the tree correspond to bootstrap values in percentages (1000 replicates). The scale bar corresponds to amino acid divergence per site between sequences. (**b**) Domains are displayed as colored boxes, and the sequence size is shown as the number of nucleotides.

The obtained amino acid sequence was aligned with 14 genomic sequences from the family *Nodaviridae*. Phylogenetic analysis based on the RdRp region indicated that *Ouro Verde nodavirus* formed a monophyletic clade with viruses that are not classified within the family *Nodaviridae* (Figure 5a).

The identity matrix constructed based on the nucleotide and amino acid alignments of the RdRp region revealed low shared identity between *Ouro Verde nodavirus* and other viruses from the family *Nodaviridae*, with *Ouro Verde nodavirus* showing values ranging from 32.7% to 48.1% for nucleotide identity and from 13.4% to 36.6% for amino acid identity, respectively (Supplementary Table S3). Analyses obtained using the InterProScan tool identified functional domains similar to those found in other species of the family *Nodaviridae* (Figure 5b).

#### 3.1.4. Iflaviridae

A sequence related to members of the family *Iflaviridae* was identified in *C. insignis* (AR886251) and was named *Carajas iflavirus* (GenBank: PQ266782). The recovered sequence exhibited 767× coverage, a length of 10,060 nt, and a complete ORF (8742 nt) that encodes a polyprotein. Analyses conducted with BlastX showed that *Carajas iflavirus* has 53.7% identity with *Budalangi Iflavi-like virus* (GenBank: UCW41643.1), a virus from the family *Iflaviridae* (Table 2).

**Figure 6:**
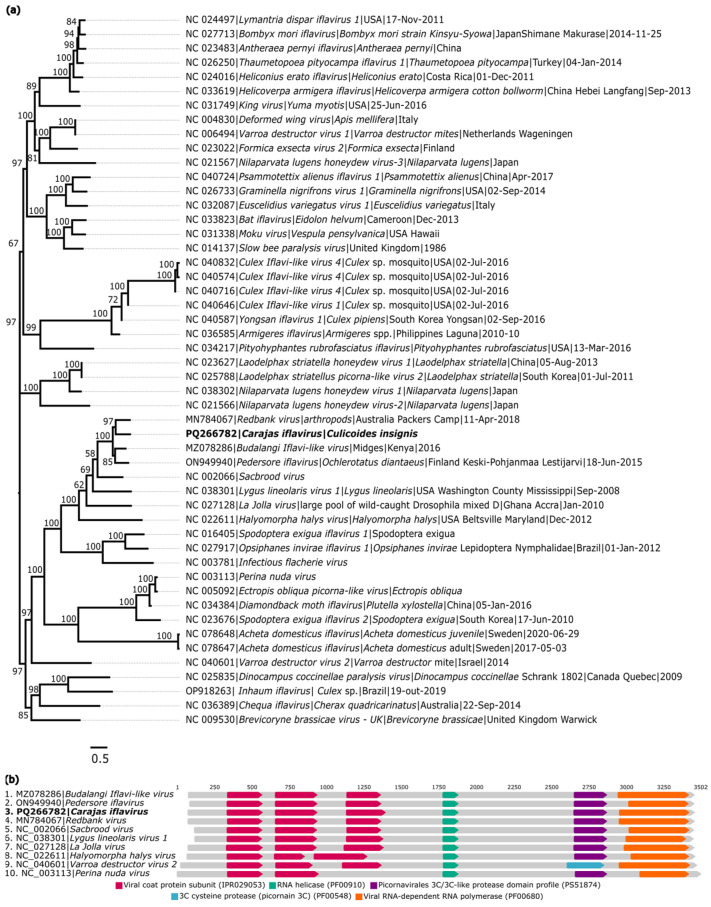
Phylogenetic relationship and genomic organization of *Carajas iflavirus*. (**a**) Phylogenetic inference was performed using the Maximum Likelihood (ML) method based on the amino acid sequences of the polyprotein, using the VT+F+R7 matrix as the best nucleotide substitution model, and measurement of the phylogenetic signal in the dataset, showing only 18% unresolved quartets and 82% resolved quartets. The sample identified in this study is highlighted in bold. The numbers at each main node of the tree correspond to bootstrap values in percentages (1000 replicates). The scale bar corresponds to amino acid divergence per site between sequences. (**b**) Domains are displayed as colored boxes, and the sequence size is shown as the number of nucleotides.

The obtained amino acid sequence was aligned with 49 genomic sequences from the family *Iflaviridae*. Phylogenetic analysis showed that *Carajas iflavirus* formed a monophyletic clade with an unclassified virus named *Redbank virus* (GenBank: MN784067) (Figure 6a).

The identity matrix generated from the nucleotide and amino acid sequence alignments showed that the obtained viral genome has low identity compared to other iflaviruses. The values for the *Carajas iflavirus* matrix ranged from 31.2% to 56.9% and from 13.2% to 51.9% for nucleotide and amino acid identity, respectively. The most significant correspondence was observed with *Budalangi Iflavi-like virus*, showing nucleotide and amino acid identities of 55.4% and 49.2%, respectively (Supplementary Table S4). Analyses obtained using the InterProScan tool identified the same functional domains present in other species of the family *Iflaviridae* (Figure 6b).

#### 3.1.5. Mesoniviridae

A contig closely related to the genus *Alphamesonivirus* (family *Mesoniviridae*) was identified in the species *C. diabolicus* (AR886257) and was named *Maraba mesonivirus* (GenBank: PQ226785). The recovered genome has 148× coverage, is 15,795 nucleotides long, and is composed of two ORFs that encode the polyprotein 1a and polyprotein 1b, with lengths of 7680 nt and 7821 nt, respectively.

BlastX analyses showed that the identified genome has 34.4% identity with *Kadiweu virus* (GenBank: YP_009666293.1), a virus from the genus *Alphamesonivirus* belonging to the family *Mesoniviridae*, referring to the polyprotein 1a region (Table 2).

**Figure 7:**
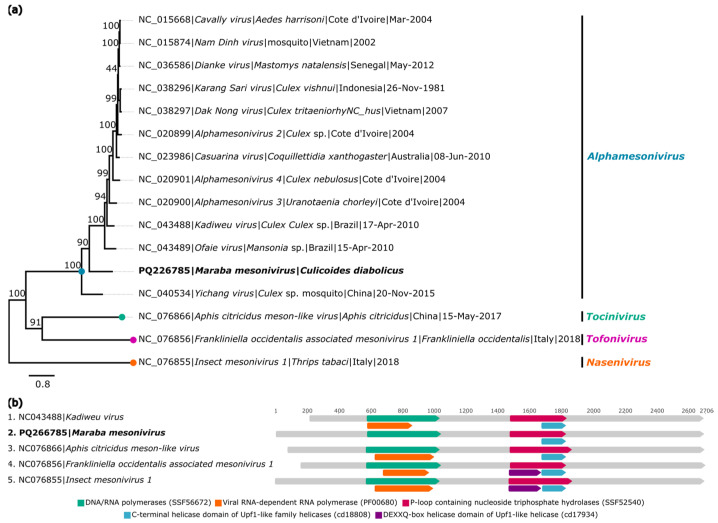
Phylogenetic relationship and genomic organization of *Maraba mesonivirus*. (**a**) Phylogenetic inference was performed using the Maximum Likelihood (ML) method based on the amino acid sequences of polyprotein 1a, using the LG+F+R5 matrix as the best amino acid substitution model, and measurement of the phylogenetic signal in the dataset, showing only 8.2% unresolved quartets and 91.8% resolved quartets. The genera are identified by different colors on the right side of the tree. The sample identified in this study is highlighted in bold. The numbers at each main node of the tree correspond to bootstrap values in percentages (1000 replicates). The scale bar corresponds to amino acid divergence per site between sequences. (**b**) Domains are displayed as colored boxes, and the sequence size is shown as the number of nucleotides.

Phylogenetic analysis showed that the fragment of polyprotein 1a obtained formed a monophyletic clade with other viruses from the genus *Alphamesonivirus* (Figure 7a). The identity matrix showed that the closest proximity was observed in relation to *Kadiweu virus*, with nucleotide and amino acid identities of 57.6% and 51.1%, respectively (Supplementary Table S5). As was performed for the previously described genome, analyses using the InterProScan tool identified functional domains typical of viruses from the family *Mesoniviridae* (Figure 7b).

#### 3.1.6. Flaviviridae

Two genomes associated with Jingmenviruses, a group of segmented viruses from the *Flaviviridae* family that are not classified at the genus level, were identified in a batch of *C. diabolicus* (AR886257) and named *Carajing-like virus 1* (GenBank: PQ226783) and *Carajing-like virus 2* (GenBank: PQ226784). The first sequence has greater completeness, showing 815× coverage, a 2706 nt length, and an ORF (2403 nt) located on segment 3 that encodes the NSP2 protein. The second sequence, with lower completeness, showed 9× coverage, a 2306 nt length, and an ORF (2289 nt) located on the same segment as the first, encoding the NSP2 protein.

BlastX analyses showed that the genomes *Carajing-like virus 1* and *Carajing-like virus 2* had 42.9% and 43.1% identities, respectively, with *Carajing virus* (GenBank: BCG55375.1), pertaining to the NSP2 region (Table 2).

**Figure 8:**
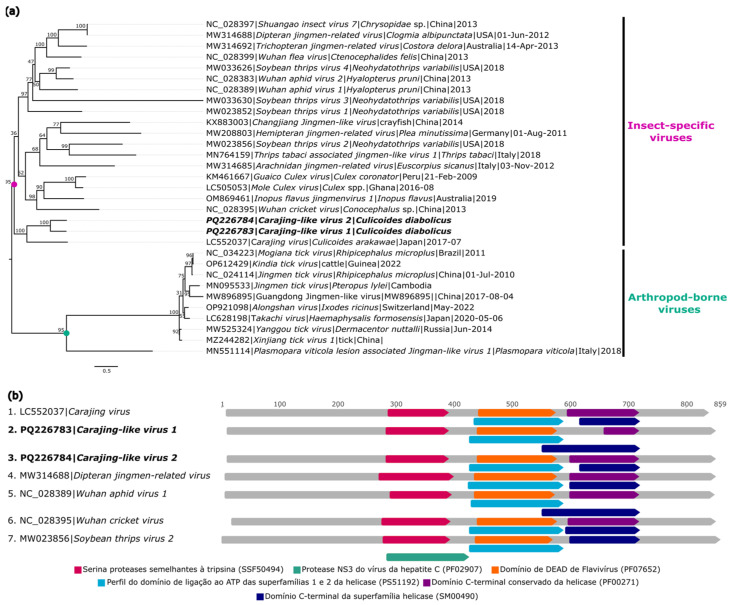
Phylogenetic relationship and genomic organization of *Carajing-like virus 1* and *Carajing-like virus 2*. (**a**) Phylogenetic inference was performed using the Maximum Likelihood (ML) method based on the amino acid sequences of the NSP2 protein, with the LG+F+R4 matrix used as the best amino acid substitution model, and the measurement of the phylogenetic signal in the dataset, showing only 17% unresolved quartets and 82% resolved quartets. The classification of viruses according to their hosts is indicated by different background colors in the tree. The samples identified in this study are highlighted in bold. The numbers at each main node of the tree correspond to bootstrap values in percentages (1000 replicas). The scale bar corresponds to amino acid divergence per site among sequences. (**b**) Domains are displayed as colored boxes, and the sequence size is shown as the number of nucleotides.

The amino acid sequences of the ORF corresponding to the NSP2 protein of the two sequences were aligned to a dataset of 29 sequences from viruses of the Jingmenvirus group, including arboviruses and arthropod-specific viruses. The monophyletic analysis showed that the obtained sequences formed a monophyletic clade with *Carajing virus*, with this clade being the most basal in the large clade of Jingmenviruses exclusively associated with arthropods (Figure 8a).

The identity matrix showed that *Carajing-like virus 1* and *Carajing-like virus 2* had nucleotide identities of 49.2% and 48.6% and amino acid identities of 41.1% and 41.6%, respectively, with *Carajing virus* (GenBank: NC_024017). Between each other, the sequences were highly divergent, with 61.2% and 60.9% nucleotide and amino acid identies, respectively (Supplementary Table S6). InterProScan analyses identified functional domains typical of viruses from the *Flaviviridae* family (Figure 8b).

#### 3.1.7. Negevirus Taxon

The genome of a new virus attributed to the *Negevirus* group was also recovered from the *C. diabolicus* (AR886257) pool, named *Maraba negevirus* (GenBank: PQ226786). The genome has 69.7× coverage, is 9941 nt in length, and has a genomic organization like that of other members of the taxon, containing three ORFs (ORF1 with 7050 nt, ORF2 with 1305 nt, and ORF3 with 705 nt) that encode hypothetical proteins 1, 2, and 3.

BlastX analyses showed that the recovered genome has 45% identity with *Turkana negevirus* (GenBank: UCW41657.1), comparing to the region of hypothetical protein 1 (Table 2).

The identity matrix showed that the virus, here referred to as *Maraba negevirus*, presented nucleotide and amino acid identities, considering the translation of the three concatenated ORFs, of 48.1% and 33.1%, respectively, with *Turkana negevirus*, described in ceratopogonids from Kenya in 2016 (Supplementary Tables S7–S9).

**Figure 9:**
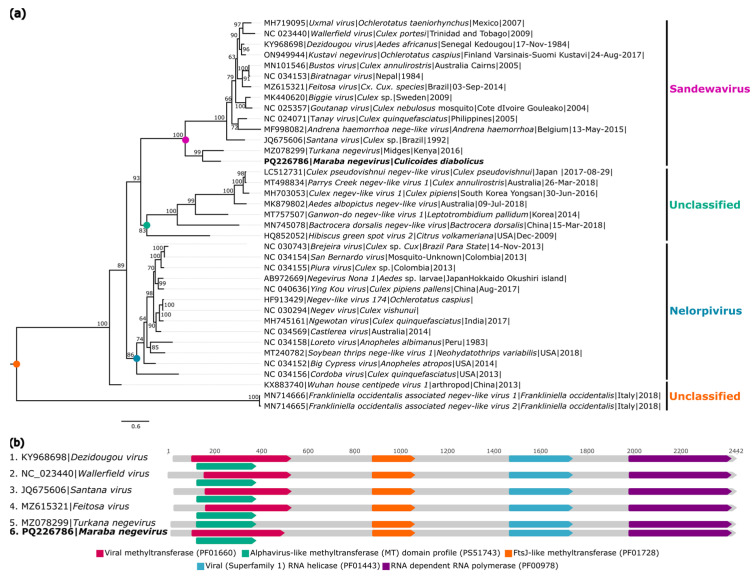
Phylogenetic relationship and genomic organization of *Turkana-like negevirus*. (**a**) Phylogenetic inference was performed using the Maximum Likelihood (ML) method based on the amino acid sequences of hypothetical protein 1, with the LG+F+I+G4 matrix used as the best amino acid substitution model, and the measurement of the phylogenetic signal in the dataset, showing only 8.2% unresolved quartets and 79.9% resolved quartets. The classification of viruses according to their hosts is indicated by the different background colors in the tree. The samples identified in this study are highlighted in bold. The numbers at each main node of the tree correspond to bootstrap values in percentages (1000 replicas). The scale bar corresponds to amino acid divergence per site among sequences. (**b**) Domains are displayed as colored boxes, and the sequence size is shown as the number of nucleotides.

The amino acid sequences corresponding to the domain of hypothetical protein 1 were aligned with 36 negevirus sequences to determine the phylogenetic relationships (Figure 9a). The analyses showed that *Maraba negevirus* is grouped with *Turkana negevirus* in a more basal subclade of viruses called Sandewavirus. The same protein domains present in other negevirus genomes were identified (Figure 9b).

## 4. Discussion

Metagenomic studies on vector insect populations are fundamentally important for the surveillance of emerging and re-emerging viruses, as well as for gaining knowledge of a variety of viral species restricted to vertebrate hosts. In this study, a high-throughput RNA sequencing approach combined with a metagenomic approach was adopted to characterize the virome of biting midges collected in the municipalities of Canaã dos Carajás, Curionópolis, and Marabá. To the best of our knowledge, this is the first investigation in Brazil that utilizes this methodology to characterize the RNA virome in biting midges identified at the species level in the Brazilian Amazon region.

Although no pathogenic viruses of medical and veterinary interest were found, 63 viral families were identified, considering the sequences with the highest numbers of reads. These families include those that harbor arboviruses such as *Flaviviridae*, *Peribunyaviridae*, *Sedoreoviridae* (formerly *Reoviridae*), *Rhabdoviridae*, and *Asfaviridae*, as well as families that include plant and/or insect-specific viruses. Thus, a variety of genomic sequences associated with viruses from a diversity of known viral families were identified.

From the bioinformatics analyses of the data generated in this study, it was found that the RNA virome composition pattern present in *Culicoides* from the three studied municipalities was divergent, suggesting that these differences may be attributed to the intrinsic characteristics of the environment in which they live [10,35,36]. The composition of the virome is the result of a complex interaction between insect species and their environment [36,37]. Therefore, it is believed that these viruses have been acquired from various sources, such as blood feeding, nectar, and the larval habitat [10,35].

A total of nine viral genomes recovered attributed to the order *Tolivirales*, the families *Chuviridae*, *Nodaviridae*, *Iflaviridae*, *Mesoniviridae*, and *Flaviviridae*, as well as to the *Negevirus* taxon were associated with ISV sequences. The *C. diabolicus* sample exhibited the highest number of genomes recovered in this study, with four different sequences. *C. leopoldoi* and *C. insignis* hosted two different genomes each.

Two genomes corresponding to the same virus, named *Ouro Verde tombus-like virus 1*, were identified in two pools of *C. leopoldoi* (AR886250; AR886254) collected in two locations in the municipality of Canaã dos Carajás: Vila Ouro Verde and the urban area. It is possible that *Ouro Verde tombus-like virus 1* is circulating in the *C. leopoldoi* population in this region, as both strains were identified in two independent pools from two locations.

*Ouro Verde tombus-like virus 1* was grouped into a clade provisionally named Gopevirus (order: *Tolivirales*), considered as a sister clade to *Tombusviridae*, which is so diverse that it suggests the designation of a new family [38]. According to these authors, the viruses grouped in this clade have already been detected in bats, spiders, and insects from the orders Zygentoma, Odonata, Hymenoptera, Megaloptera, Neuroptera, Coleoptera, Raphidioptera, and Diptera. The data from this investigation showed that the two strains of *Ouro Verde tombus-like virus 1* are grouped in a subclade of viruses associated with wasps. However, despite having a higher identity with *Hymenopteran tombus-related virus*, the nucleotide and amino acid identities of the genome identified in this study were low compared to other registered viruses belonging to this clade, indicating that *Ouro Verde tombus-like virus 1* represents a new viral species, being the first record of Gopevirus in *Culicoides* and the first finding of a viral species from this clade in Brazil and the Brazilian Amazon region.

Another ISV identified in samples of the species *C. leopoldoi* from the urban area of Canaã dos Carajás was associated with the family *Chuviridae*. Viruses in this family have an RNA genome containing two to four ORFs and have been associated with arachnids, barnacles, crustaceans, fish, reptiles, and insects from the orders Odonata, Dermaptera, Blattaria, Coleoptera, Hemiptera, Hymenoptera, and Diptera in Africa, Asia, Australia, Europe, North America, and South America [39]. 

The data from this study based on the recovered RdRp region showed that the *Carajas chuvirus* did not group with any of the genera attributed to the family *Chuviridae*. However, the obtained data demonstrate an ancestral relationship between the identified virus and other members already described in this family. Although it did not group with the genera assigned to *Chuviridae*, it was found that the *Carajas chuvirus* was more closely related to the genera *Piscichuvirus* and *Chuvivirus*, which include viruses associated with atheriniform and silverside fish, scaly reptiles, and decapod crustaceans. Additionally, unclassified viruses related to the genus *Chuvivirus* were found in insects from the orders Diptera, Coleoptera, Hemiptera, Odonata, and Orthoptera [39]. Despite the *Carajas chuvirus* showing phylogenetic relation and the same protein domains found in other viruses already identified in the family *Chuviridae*, the nucleotide and amino acid identities of this virus were low, indicating that the virus identified in this study is a new species in the family *Chuviridae*.

Other Chuviviruses identified in *Culicoides* have already been recorded on the Asian continent in the species *C. impunctatus* [17] and on the European continent in *Culicoides cataneii* Clastrier, 1957 and *Culicoides haranti* Rioux, Descous & Pech, 1959 [40]. In Brazil, viruses from the family *Chuviridae* have already been found in dipterans of the family Culicidae, including *Mansonia wilsoni* Barreto & Coutinho, 1944, *Psorophora dimidiata* Cerqueira, 1943, *Psorophora pseudomelanota* Barata & Cotrim, 1971, and *Stegomyia albopicta* Skuse, 1894 in the Cerrado [41]; *Sabethes gymonothorax* Harbach & Petersen, 1922 in the Pantanal of Mato Grosso [42]; and in *M. wilsoni* and *Coquillettidia hermanoi* Lane & Coutinho, 1940 in the Atlantic Forest, Recife, Pernambuco [43]. This is the first detection of viruses from the family *Chuviridae* in dipterans in the Brazilian Amazon region and the first identification in *Culicoides* in Brazil.

In this investigation, two genomes related to ISVs were identified in the species *C. insignis*, collected in Vila Ouro Verde, in the municipality of Canaã dos Carajás. This species, frequently associated with cattle, is considered an important vector of *Orbivirus caerulinguae* in Central and South America. This virus causes Bluetongue, an infectious disease that affects both wild and domestic ruminants, resulting in serious socioeconomic and sanitary impacts [12].

The genomes identified in *C. insignis* were associated with two families: *Nodaviridae* and *Iflaviridae*. The family *Nodaviridae* has two genera, *Alphanodavirus* and *Betanodavirus* [44]. In this study, the recovered ISV grouped into a clade of unclassified viruses from this family, more closely related to viruses attributed to the genus *Alphanodavirus*, whose members are associated with insects from the orders Diptera, Lepidoptera, Dermaptera, and Odonata [45]. It was found that the clade in which the *Ouro Verde nodavirus* identified in this study was grouped also includes the *Carano virus*, a virus identified in the species *Culicoides arakawae* Arakawa, 1910 in Tokyo, Japan [46]. Additionally, the recovered genome showed the presence of the same protein domains found in other nodaviruses. However, the low nucleotide and amino acid identities of the *Ouro Verde nodavirus* compared to other viruses already recorded in the family *Nodaviridae* indicate that the identified genome is a new species in this family.

In Brazil, viruses from the family *Nodaviridae* have already been detected in the mosquito species *Culex quinquefasciatus* Say, 1823 and *Aedes aegypti* Linnaeus, 1762 in the state of Mato Grosso [47]. Therefore, this is the first record of viruses from the family *Nodaviridae* in dipterans in the core of the Brazilian Amazon region and the first detection in species of the genus *Culicoides* in Brazil.

The family *Iflaviridae* has a single genus called *Iflavirus*, in which all members have been identified in arthropods, mainly in insects [48]. In the present study, the identified ISV called *Carajas iflavirus* grouped into a clade with a virus found in an arthropod host and was more closely related to the clade that includes an *Iflavirus* called *Budalangi iflavi-like virus* identified in *Culicoides leucostictus* Kieffer, 1911 in Kenya [49]. Furthermore, the identified genome presented the same protein domains found in other viruses already registered in the family *Iflaviridae*. Despite the close relationship of the *Carajas iflavirus* with other Iflaviruses, the low nucleotide and amino acid identities of these viruses indicate that the genome identified in this study is a new species of virus in this family.

Globally, besides the *Budalangi iflavi-like virus* identified in *C*. *leucostictus* in Kenya [38], another *Iflavirus* called *Culicoides iflavirus 1* was found in *Culicoides* in China [50]. In Brazil, species of Iflaviruses have already been found in mosquitoes of the species *Ae. aegypti* in the state of Amapá [51], in *Psorophora albigenu* Peryassú, 1908 [42], *Cx. quinquefasciatus* and *Ae. aegypti* [47] in the state of Mato Grosso, and in *Culex (Culex)* spp. in the state of Maranhão [8]. Therefore, this is the first virus from the family *Iflaviridae* identified in specimens of the genus *Culicoides* in Brazil.

In this investigation, four genomes related to ISVs from the families *Mesoniviridae* and *Flaviviridae* and the taxon *Negevirus* were also identified in *C. diabolicus* from the Flona de Tapirapé-Aquiri, in the municipality of Marabá.

Regarding the family *Mesoniviridae*, it comprises the genera *Alphamesonivirus*, *Nacenivirus*, *Tocinivirus*, and *Tofonivirus*, most of which infect insects, mainly mosquitoes [52]. The genome recovered in the present study, named *Maraba mesonivirus*, was attributed to the genus *Alphamesonivirus*, grouping as a sister clade to other clades consisting of viruses found in mosquitoes of the genera *Aedes* Meigen, 1818, *Coquillettidea* Dyar, 1905, *Culex* Linnaeus, 1758, *Mansonia* Blanchard, 1901, *Uranotaenia* Lynch Arribálzaga, 1891, and in a rodent [53,54,55,56]. Additionally, the same protein domains found in other viruses of the family *Mesoniviridae* were detected. Despite the similarities, the low nucleotide and amino acid identities indicate that the recovered genome is a new species for this family.

In Brazil, a genome attributed to the family *Mesoniviridae* was previously identified in a pool of unidentified ceratopogonids at the species level, also in the Flona de Tapirapé-Aquiri, in the municipality of Marabá [57]. The study suggests that *C. diabolicus* may be involved in the circulation of this virus in this locality. Other studies identified genomes belonging to *Mesoniviridae* in *Culex* sp. and *Mansonia* spp. in the state of Mato Grosso do Sul [55] and in *Ae. aegypti* and *Cx. quinquefasciatus* in the state of Mato Grosso [32].

The family *Flaviviridae* consists of the genera *Hepacivirus*, *Orthoflavivirus*, *Pegivirus*, and *Pestivirus* [58]. However, the two genomes recovered in this study were associated with an unclassified group within the family *Flaviviridae* called *Jingmenvirus*. This group consists of a phylogenetic clade that includes viruses associated with ticks and vertebrates, and a second clade that includes Jingmenviruses associated with insects [59]. The data indicate that the genomes found in *C. diabolicus* belong to the phylogenetic clade composed of viruses that exclusively infect arthropods, forming a subclade more closely related to the *Carajing virus*, a virus identified in the species *C. arakawae* in Japan [46].

Additionally, the two identified genomes presented the same protein domains found in other Jingmenviruses. However, the low nucleotide and amino acid similarities between the two genomes compared to other members of the group indicate that *Carajing-like virus 1* and *Carajing-like virus 2* are new viruses of the family *Flaviviridae*.

Apart from Jingmenviruses, other viruses attributed to the family *Flaviviridae* were identified in species of the genus *Culicoides*. Examples include the *Circunscriptus flavi-like virus*, found in *Culicoides circunscriptus* Kieffer, 1918 in the geographical area of Thrace, southeastern Europe [40], and the *Binh virus*, identified in unspecified *Culicoides* species in Zhoushan Island, China [50]. In addition to these ISVs, RNA from the arbovirus *Orthoflavivirus nilense* was also detected in the species *Culicoides arboricola* Root & Hoffman, 1737, *Culicoides biguttatus* Coquillett, 1901, and *Culicoides stellifer* Coquillett, 1901 collected in Louisiana, USA [60]. In Brazil, as is the case worldwide, most viruses of the family *Flaviviridae*, especially from the genus *Orthoflavivirus*, have been primarily identified in mosquitoes of the genera *Aedes*, *Culex*, and *Anopheles* Meigen, 1818. These viruses have the capacity to infect humans and animals, notably including *Orthoflavivirus denguei*, *Orthoflavivirus zikaense*, *Orthoflavivirus flavi*, *Orthoflavivirus nilense*, and *Orthoflavivirus ilheusense* [61,62,63,64,65]. So far, no viruses from the family *Flaviviridae* have been identified in *Culicoides* in Brazil, making this the first record of a virus associated with this family in these dipterans in the country.

Viruses of the *Negevirus* taxon have a wide geographical distribution and infect a variety of blood-feeding insects, such as mosquitoes from the genera *Culex*, *Aedes*, and *Anopheles*, as well as phlebotomine sandflies from the genus *Lutzomyia* França, 1924 [66].

The metagenomic analyses conducted in this investigation showed that the identified genome was attributed to the *Sandewavirus* clade, grouping with the *Turkana virus*, which is found in various species of *Culicoides* in Kenya [49]. Additionally, the genome identified in this study showed low nucleotide and amino acid similarity to other Negeviruses already recorded, indicating that it is a new virus species named *Maraba negevirus*. The presence of the same protein domains found in genomes of other viruses belonging to this taxon was also observed.

In Brazil, Negeviruses have been identified in the mosquitoes *Cx. quinquefasciatus* and *Ae. aegypti* in the state of Mato Grosso [47]. In the Amazon region, 14 strains of ISVs from the *Negevirus* taxon were detected in *Cx. (Cux.) coronator* Dyar & Knab, 1906 and *Culex* sp., with 13 found in the municipality of Canaã dos Carajás and 1 found in the municipality of Curionópolis [67]. Therefore, *Maraba negevirus* is the first *Negevirus* identified in *Culicoides* in Brazil.

The results of this study revealed the viral diversity present in species of *Culicoides*, a frequently overlooked genus that harbors several species associated with the transmission of arboviruses causing diseases in humans and animals worldwide [12]. In Brazil, between January and June 2024, the Ministry of Health reported 6973 confirmed cases of Oropouche Fever, representing 84.2% of the total 8284 cases recorded between 2015 and 2023 [21]. Furthermore, species of the genus *Culicoides* are implicated in the transmission of various arboviruses, such as *Orthobunyavirus schmallenbergense*, *Orthobunyavirus shuniense*, *Orthobunyavirus ainoense*, *Orbivirus caerulinguae*, *Orbivirus ruminantium*, *Orbivirus alphaequi*, and *Ephemerovirus febris*, which affect a variety of animals, including sheep, goats, cattle, buffalo, deer, and horses [11,12,13,14,15,16].

This information underscores the importance of this genus as a potential vector of arboviruses and highlights the ongoing need for entomovirological surveillance. Such research is essential for understanding the viral diversity present in species of the genus Culicoides and for assessing public health risks, especially regarding the possibility of spillover and outbreaks of diseases caused by viruses transmitted by biting midges.

Metagenomics serves as an essential tool in the study of viromes, revealing a remarkable number of new viruses, many of which are not cultivable. With this tool, it has been possible to obtain information and evidence about the RNA viral diversity circulating in species of the genus *Culicoides* in the influence area of the Carajás mineral complex in the state of Pará, the Brazilian Amazon region. This demonstrates the capacity of species in this genus, which is less studied compared to other dipterans, to harbor unknown viruses.

## 5. Conclusions

Finally, this study presents the first characterization of the virome in *Culicoides* species in Brazil, a genus that includes vector species of viruses that cause diseases in humans and animals. Future studies investigating viral diversity in *Culicoides*, as well as that explore topics such as population genetics, hostpathogen interactions, and vector competence, are essential, given the relevance of this genus to public health.

## Figures and Tables

**Table 1:** Descriptions of biting midge samples used for metagenomic analysis and data generated.

Sample	Species	Number of Specimens	Locations	Collection Date	Raw Reads	Reads After Fastp Treatment	Reads After SortMe RNA Treatment
AR886250	*Culicoides leopoldoi* Ortiz, 1951	150	VOV ^1^	13–16 February 2019	40,506,952	34,662,964	27,390,562
AR886251	*Culicoides insignis* Lutz, 1913	150	VOV ^1^	13–16 February 2019	46,412,998	43,216,228	27,561,613
AR886252	*Culicoides limai* Barretto, 1944	150	VOV ^1^	13–16 February 2019	44,295,920	41,073,206	23,198,834
AR886253	*Culicoides paucienfuscatus* Barbosa, 1947	153	SP ^2^	20–27 February 2019	45,635,832	39,495,690	38,572,298
AR886254	*Culicoides leopoldoi* Ortiz, 1951	150	UA ^1^	8–11 February 2019	44,345,628	36,679,300	28,354,130
AR886255	*Culicoides pseudodiabolicus* Fox, 1946	150	NFCC ^1^	4–11 April 2019	46,999,790	42,441,708	30,247,110
AR886256	*Culicoides foxi* Ortíz, 1950	86	NFCC ^1^	4–11 April 2019	55,096,898	52,158,522	41,950,709
AR886257	*Culicoides diabolicus* Hoffman, 1925	150	TANF ^3^	23 February–4 March 2019	50,600,182	46,103,744	31,117,695
Total	-	1139	-	-	373,894,200	335,831,362	248,392,951

^1^ Canaã dos Carajás: VOV—Vila Ouro Verde (S 06°31′38.2″; W 50°08″52.5″); AU—urban area (S 06°31′08.1″; W 49°51′18.8″); NFCC—National Forest Canaã dos Carajás (S 06°26′24.8″; W 50°19′37.2″). ^2^ Curionópolis: SP—Serra Pelada (S 05°56′35.3″; W 049°40′38.7″). ^3^ Marabá: NFTA—National Forest Tapirapé-Aquiri (S 05°46′16,4″; W 50°33′15.8″).

**Table 2:** Viral sequences obtained from *Culicoides* in the municipalities of Canaã dos Carajás, Curionópolis, and Marabá, in the state of Pará, Brazil, and their best results compared to the non-redundant (nr) database from NCBI using BlastX.

Virus (GenBank Accession)	Sample/ Host	Recovered Genome (nt)	Average Coverage	Classification	Closest Viruses/ Access to GenBank	BlastX			
						Region	Amino Acid Identity (%)	QC ^1^ (%)	E-Value
*Carajas tombus-like virus 1*	AR886250/ *C. leopoldoi*	2163	78	*Tombusviridae*	*Hymenopteran tombus-related virus*/QTJ63591.1	RdRp ^2^	40.8	99	8 × 10^31^
*Carajas tombus-like virus 1*	AR886254/ *C. leopoldoi*	1166	73.9	*Tombusviridae*	*Hymenopteran tombus-related virus*/QTJ63591.1	RdRp ^2^	40.1	99	8 × 10^31^
*Ouro verde nodavirus*	AR886251/ *C. insignis*	3213	803	*Nodaviridae*	*Carano virus*/BCG55383.1	RdRp ^2^	51.8	87	0
*Carajas iflavirus*	AR886251/ *C. insignis*	10,060	767	*Iflaviridae*	*Budalangi Iflavi-like virus*/UCW41643.1	Pol ^3^	53.7	96	0
*Carajas chuvirus*	AR886254/ *C. leopoldoi*	5827	53.4	*Chuviridae*	*Chuviridae* sp./ UCR92571.1	RdRp ^1^	58.3	59	0
*Maraba mesonivirus*	AR886257/ *C. diabolicus*	15,795	148	*Mesoniviridae*	*Kadiweu virus*/YP_009666293.1	Pol 1a ^4^	34.4	86	0
*Maraba negevirus*	AR886257/ *C. diabolicus*	9941	69.7	*Negevirus*	*Turkana negevirus*/MZ078299.1	HP 1 ^5^	45	46	0
*Carajing-like virus 1*	AR886257/ *C. diabolicus*	2706	815	*Flaviviridae*	*Carajing virus*/BCG55375.1	NSP2 ^6^	42.9	92	0
*Carajing-like virus 2*	AR886257/ *C. diabolicus*	2306	9	*Flaviviridae*	*Carajing virus*/BCG55375.1	NSP2 ^6^	43.1	96	0

^1^ Query cover; ^2^ RNA-dependent RNA polymerase; ^3^ polyprotein; ^4^ polyprotein 1a; ^5^ hypothetical protein 1; ^6^ non-structural proteins 2.

## Data Availability

The raw sequence reads generated in this study are available at the NCBI Sequence Read Archive (SRA) database under BioProject PRJNA1108159 and BioSamples SAMN41238809, SAMN41238810, SAMN41238813, and SAMN41238816. All virus contigs generated in this study have been deposited in GenBank under accession numbers: PQ226781 to PQ226789.

## Supplementary Materials

The following supporting information can be downloaded at https://www.mdpi.com/article/10.3390/v16121862/s1, Table S1: Nucleotide and amino acid identity matrix of *Ouro verde tombus-like virus 1* (AR886250) and *Ouro verde tombus-like virus 1* (AR886254) compared with members of the more closely related Gopeviruses, Suriviruses, and Aspoviruses clades; Table S2: Nucleotide and amino acid identity matrix of *Carajas chuvirus* compared with more closely related members of the family *Chuviridae*; Table S3: Nucleotide and amino acid identity matrix of *Ouro verde nodavirus* compared with more closely related members of the family *Nodaviridae*; Table S4: Nucleotide (nt) and amino acid (aa) identity matrix of *Carajas iflavirus* compared with more closely related members of the *Iflaviridae* family; Table S5: Nucleotide (nt) and amino acid (aa) identity matrix of *Maraba mesonivirus* compared with more closely related members of the *Mesoniviridae* family; Table S6: Nucleotide (nt) and amino acid (aa) identity matrix of *Carajing-like virus 1* and *Carajing-like virus 2* compared with members of the most closely related *Jingmenvirus* group (*Flaviviridae*); Table S7: Matrix of amino acid and nucleotide identities based on alignments corresponding to ORF 1 of the viruses phylogenetically closest to *Maraba negevirus*; Table S8: Matrix of amino acid and nucleotide identities based on alignments corresponding to ORF 2 of the viruses phylogenetically closest to *Maraba negevirus*; Table S9: Matrix of amino acid and nucleotide identities based on alignments corresponding to ORF 3 of the viruses phylogenetically closest to *Maraba negevirus*.
